# Influence of the Exercise-psychology Adjustment Mode on the Mental Health of Medical Workers

**Published:** 2017-06

**Authors:** Wenxin XU, Mengjuan CENG, Jiwei YAO, Longfei CHEN

**Affiliations:** 1. Institute of Physical Education and Sport Science, Fujian Normal University, Fuzhou, Fujian, 350117, China; 2. Physical Education College, Hunan University of Science and Technology, Xiangtan, Hunan, 411201, China; 3. The First Affiliated Hospital Fujian Medical University, Fuzhou, Fujian, 350005, China

**Keywords:** Medical workers, Mental health, Exercise–psychology adjustment mode, Intervention

## Abstract

**Background:**

Chinese medical workers suffer from a high incidence of mental health problems, resulting in reduced efficiency, increased medical malpractice, rising medical costs, and other issues. The effective alleviation of mental health problems among medical workers is therefore an important focus of research and social attention.

**Methods:**

The mental health of 842 medical workers from the First Affiliated Hospital of Fujian Medical University and the Second Affiliated Hospital of Fujian Medical University in Fuzhou, China was evaluated between February 2016 and March 2016. Sixty-two workers with positive SCL-90 screening results were selected as the subjects to be investigated in the intervention experiment, with 31 in the intervention group and 31 in the control group. The control group did not participate in any regular physical exercise activity for the 4-month duration of the study, whereas the exercise–psychology adjustment mode was applied to the intervention group.

**Results:**

Medical workers had a higher total SCL-90 score and number of positive items than the national norm (*P* < 0.05). After the intervention, the SCL-90 score, number of positive results, somatization, obsessive-compulsive symptoms, sensitivity, depression, anxiety, and hostility of the intervention group were significantly lower than they were before the intervention (*P* < 0.05) and lower than those of the control group (*P* < 0.05).

**Conclusion:**

The alleviation of mental health problems, which are increasingly serious among medical workers, should be a matter of societal focus. The exercise–psychology adjustment is an effective intervention mode for the mental health of medical workers.

## Introduction

Medical workers are a special occupational group. On the one hand, they need to help patients to get rid of physical and mental illnesses and restore a healthy life; on the other hand, medical workers also personally have a variety of physical and psychological needs. Medical workers’ mental health can be disturbed because of long-term exposure to a highly stressful working environment and complex doctor–patient relationships. In fact, the mental health problems of medical workers have always been the concern of many psychologists.

One researcher (1) examined the working conditions and mental health status of 1505 female medical workers in the UK, revealing a low level of mental health in this demographic. Among the factors that affect the mental health of medical workers, work stress, psychological burden, and lack of sleep have been reported to be important variables. Lower sleep quality and lower mental health levels of Japanese nurses increased the probability of the occurrence of medical malpractice (2). Wang et al. (3) conducted a mental health survey targeted at 2460 medical workers in Beijing using the Symptom Check List; the results showed that the levels of mental health among medical workers were lower than the national norm, with a relatively high psychological problem detection rate, and that gender, age, department, and other factors affected the mental health of medical workers. The mental health problems of medical workers are not only related to the physical and mental health of medical workers themselves, but also associated with improvement and enhancement of the overall psychological quality of the medical team as well as the promotion of the living quality of the societal population. Therefore, it is of great practical significance to pay attention to the mental health of medical workers and to formulate targeted and flexible mental health adjustment programs for these workers.

Physical exercise is quite beneficial to health and more and routine exercises have been used in the early prevention, mid-term treatment, and late recovery of some major chronic diseases, such as cancer and cardiovascular diseases (4–5). With the deepening of the research, physical exercise has also been used in interventions for mental health and psychological well-being (6–7). After applying a physical exercise intervention in the treatment of depression, in both the intervention group and the control group, after adhering to physical exercise for some time, the levels of individual depression were significantly lower than the figures before the intervention (8). Scheewe et al. (9) examined the effects of physical exercise and drug therapy on the mental and physical health of patients with schizophrenia. After 6 months of intervention twice a week, the mental and physical health levels of patients in the physical exercise group were significantly improved, while there was little change in the drug therapy group.

The treatment effects of physical exercise on anxiety and other psychiatric disorders have also been documented (10–11). The primary features of physical exercise as an intervention approach are flexibility and pertinence. Different individuals can choose appropriate exercises, modes of exercise, and exercise times according to their own situation. Since medical workers are required to bear high workloads within a short time, this mental health intervention mode is quite suitable for them. Zhang (12) used these characteristics of exercise intervention to intervene in the mental health of medical school students, forming a mental health self-exercise intervention mode featuring “exercise prescription, mental health, and self-adjustment.” After 13 weeks of intervention, the mental health of those medical students was significantly improved.

While the studies above provide a useful reference for intervention in the mental health of medical workers, they were mostly conducted from the perspective of the tester. For one thing, the intervention plans were not targeted, since they neither varied from person to person nor took into account the adjustment effects of different exercise modes on different psychological conditions. Additionally, as experimental subjects, the medical workers lacked initiative, resulting in weak persistence of the intervention effects.

To address these problems, in this study, we developed a self-adjustment mode based on the combination of exercise and psychology and use appropriate exercise modes to intervene in medical workers’ mental health according to different psychological conditions, giving full play to their subjective initiative. Through this process, we construct the “exercise–psychology” adjustment mode—a self-intervention mode—in order to provide new ideas and methods for promoting the physical and mental health of medical workers.

## Methods

A total of 842 medical workers were selected from the First Affiliated Hospital of Fujian Medical University and the Second Affiliated Hospital of Fujian Medical University in Fuzhou, China to complete the Symptom Checklist 90 (SCL-90) between February and March 2016. In the second phase, from April to August 2016, 62 workers with positive SCL-90 screening results were involved in the intervention, which lasted 4 months. All subjects met the following inclusion criteria: 1) aged between 18 and 60; 2) physical healthy and able to do exercises in the adjustment mode; 3) no mental disorders; 4) personally signing a written informed consent form; 5) one year or longer clinical working experience in a hospital. In addition, the subjects volunteered to participate in this experiment based on understanding the purpose, content, and methodology of this study. The researchers also obtained the consent and support of the units in which the subjects were working. Therefore, the study was free of any ethical issues.

The 62 participants were randomly divided into the intervention group and the control group, with 31 people in each group. The difference in gender, age, length of service, department, profession, marital status, or other basic information between the groups was not statistically significant. The intervention group took part in the adjustment intervention experiment, while the control group did not partake in the intervention experiment. The workers in both groups worked and lived according to their normal routine. After the experiment, the SCL-90 and the Simplified Coping Style Questionnaire (SCSQ) were used to test the subjects.

### Investigation Method

Group testing was the investigation method used in the first phase, mainly conducted among medical workers during the morning shift or relatively concentrated time. The SCL-90 questionnaire (13–15) was employed as the investigation tool. The SCL-90 contains 90 items rated on a scale from 1 to 5 points, resulting in a total score between 90 and 450 points. The total score reflects the severity of the disease. Specifically, a positive screening result is determined by a total score of more than 160 points, more than 43 positive items, or a score exceeding 2 on any factor (13–15). Our version of the questionnaire also covered gender, age, length of service, department, profession, and other demographic items.

The researchers consisted of two postgraduates of psychology and three PhDs and professors in the field of sports. They were able to provide reliable quality assurance for the proper implementation of this study. The questionnaire was distributed by the trained investigators and completed by the participants voluntarily under unified guidance. The investigation data were compared to the national norm (16) and analyzed.

### Intervention Method

1) Assessment. Before the implementation of the exercise–psychology adjustment mode, the subjects needed be exposed to and master relevant knowledge of the mode to provide a theoretical basis for its formulation and implementation. The researchers used roughly two days of rest time to explain the composition, implementation, and precautions of the adjustment mode to the subjects. The specific contents were: 1) the composition of the exercise–psychology adjustment mode, including setting the exercise target, preparing, selecting the exercise item, determining the exercise frequency, deciding the intensity and duration of the exercise, and finishing and relaxing; 2) determining the program of the adjustment mode according to the different conditions of different people, that is, determining the program according to individual needs and objectives; 3) understanding the psychological adjustment effects of different exercise programs and leading the subjects to learn how to choose reasonable exercise items in accordance with their own psychological status, health status, physical fitness, purpose, and interest.

2) Implementation. Firstly, the subjects were tested with the SCL-90 questionnaire. The researchers collected the statistical data and informed each participant of the results, letting them know about their own mental state and providing evidence for the program development in the next step. Secondly, based on the acquisition of theoretical knowledge on exercise programs, the subjects were allowed to develop exercise prescriptions according to their own psychological symptoms. In this step, the researchers played the role of assisting and providing guidance to amend and improve the exercise prescriptions developed by the subjects, especially in the determination of the sport item, exercise intensity, and exercise load, but they avoided too much intervention to eventually achieve the purpose of mastering the skill of making reasonable exercise adjustments according to different psychological symptoms. Finally, during the implementation of the proposed exercise program, the subjects were required to carry out exercise by following the program standards and steps.

3) Evaluation. In the implementation of the exercise–psychology adjustment mode, the subjects were required to write a weekly exercise record in order to establish confidence and increase motivation for the next step. After 4 months, with a month as a training cycle, the subjects were tested via the SCL-90 to evaluate the exercise effects and correct their exercise program according to the exercise effects and their own exercise experience with help of the teachers. The SCL-90 test was carried out after 4 months of exercise adjustment. The scores of each factor before and after the implementation of the exercise– psychology mode were compared to verify the effects of the intervention.

## Results

### Basic Information

A total of 842 copies of the questionnaire were distributed in the first stage, and 801 valid copies of the questionnaire were obtained, with a valid rate of 95.1%. Among the participants, there were 249 (31.1%) male workers and 552 (68.9%) female workers; 375 (46.82%) were doctors and 426 (53.18%) were nurses; 191 (23.85%) workers were employed in the area of internal medicine, 139 (17.35%) in surgery, 142 (17.73%) in gynecology and obstetrics, 138 (17.23%) in pediatrics, 140 (17.48%) in the emergency department/intensive care unit, and 50 (6.24%) in other departments. Subjects were aged 20 to 62 years old (*M* = 34.3 ± 7.9) with 1 to 39 years of service (*M* = 12.2 ± 6.1).

### Statistical Methods

The data were analyzed by SPSS 15.0 statistical software (Chicago, IL, USA). Quantitative data were presented as mean ± standard deviation. The independent *t* test was used to compare quantitative data between the two groups. The paired *t* test was adopted to compare quantitative data within a group. Comparison between several groups of quantitative data was carried out using single-factor analysis of variance (ANOVA). The number of cases and composition ratio were used to represent the qualitative data. Cross-group comparison was conducted using the chi-square test or Fisher’s exact probability method, with *P* < 0.05 as a statistically significant difference.

### Ethical Principles

The distribution of the questionnaire strictly adhered to the principle of informed consent and obtained the permission of the Fujian Medical University Ethics Committee. The researchers introduced the purpose, methodology, and principle of confidentiality to the participants, ensuring that the participants participated in the investigation voluntarily.

### Mental Health Status of Medical Workers

The analysis of the mental health status of the 801 medical workers screened out 72 subjects with total scores of more than 160 points or with more than 43 positive items; hence, the positive rate was 8.99%. The average SCL-90 score of the medical workers was significantly higher than the national norm (*P* < 0.05). The average number of positive items was also significantly higher than the national norm (*P* < 0.05). In terms of individual factors, the scores on obsessive-compulsive symptoms, sensitivity, depression, anxiety, and phobia were significantly higher than the national norm (*P* < 0.05), while the scores on somatization, hostility, paranoia, and psychosis did not differ significantly from those of the national norm (*P* > 0.05) (Table 1).

**Table 1:** Comparison between the scl-90 scores of medical workers in Fuzhou and the national norm

**Item**	**Medical workers (n = 801)**	**National norm (n = 1388)**	***t***	***P***
SCL-90 score	135.24 ± 32.12	128.96 ± 38.76	3.879	0.000
Number of positive items	31.88 ± 15.32	24.92 ± 18.41	9.040	0.000
Somatization	1.38 ± 0.51	1.37 ± 0.48	0.459	0.647
Obsessive-compulsive symptoms	1.79 ± 0.71	1.62 ± 0.58	6.073	0.000
Sensitivity	1.71 ± 0.58	1.65 ± 0.61	2.256	0.024
Depression	1.58 ± 0.61	1.5 ± 0.59	3.017	0.003
Anxiety	1.48 ± 0.54	1.39 ± 0.43	4.285	0.000
Hostility	1.54 ± 0.53	1.46 ± 0.55	0.830	0.407
Phobia	1.31 ± 0.39	1.23 ± 0.41	4.474	0.000
Paranoia	1.42 ± 0.51	1.43 ± 0.57	0.410	0.682
Psychosis	1.27 ± 0.39	1.29 ± 0.42	1.101	0.271

### Analysis of the Intervention Effect of the Exercise*–*Psychology Adjustment Mode

The 72 medical workers screened out due to a total score of more than 160 points or more than 43 positive items were asked to give their opinion. Finally, 62 subjects voluntarily participated in the post-study of intervention effect evaluation. The subjects were sequenced in a line with the serial numbers of their questionnaires and presented with a random number, based on which they were randomly divided into the intervention group and the control group (31 subjects in each group). The basic information of the two groups is shown in Table 2.

**Table 2:** Comparison of Basic Information between the Intervention Group and the Control Group

**Variable**		**Intervention group (n = 31)**	**Control group (n = 31)**	***t or χ^2^***	***P***
Age (yr)		36.5 ± 4.5	35.5 ± 4.7	0.856	0.396
Length of service		13.2 ± 3.5	14.1 ± 3.2	1.057	0.295
Gender	Male	10	11	0.072	0.788
	Female	21	20		
Profession	Doctor	13	14	0.066	0.798
	Nurse	18	17		
Department	Internal medicine	7	8	0.872	0.059
	Surgery	4	3		
	Gynecology and obstetrics	6	5		
	Pediatrics	7	7		
	Emergency dept./ICU	7	6		
	Other departments	0	2		

It can be seen from Table 2 that there was no significant difference between the intervention group and the control group in SCL-90 score, number of items, or score on each factor before the intervention (*P* > 0.05), meaning that the basic information of the two groups was comparable.

After the intervention, the average SCL-90 score, number of positive items, and scores on somatization, obsessive-compulsive symptoms, sensitivity, depression, anxiety, and hostility of the intervention group were significantly lower than those of the same group before the intervention (*P* < 0.05) and those of the control group after the intervention (*P* < 0.05). The scores on the three factors of phobia, paranoia, and psychosis were also lower than those of the intervention group before the intervention and the control group after the intervention, but the differences were not significant (*P* > 0.05) (Table 3).

**Table 3:** Analysis of the intervention effect of the exercise–psychology adjustment mode

**Item**		**Before intervention**	**After intervention**	***t^#^***	***P^#^***
SCL90 score	Intervention group	175.24 ± 25.12	155.96 ± 27.58	2.430	0.018
	Control group	173.29 ± 25.78	171.12 ± 28.76	0.601	0.550
	t^*^	0.456	2.118		
	P^*^	0.650	0.038		
Number of positive items	Intervention group	45.88 ± 16.32	33.12 ± 18.41	2.254	0.028
	Control group	44.12 ± 17.35	42.92 ± 18.77	0.261	0.795
	t^*^	0.411	2.075		
	P^*^	0.682	0.042		
Somatization	Intervention group	1.71 ± 0.53	1.35 ± 0.51	2.725	0.008
	Control group	1.67 ± 0.54	1.62 ± 0.54	0.365	0.717
	t^*^	0.294	2.024		
	P^*^	0.770	0.047		
Obsessive-compulsive symptoms	Intervention group	2.14 ± 0.75	1.75 ± 0.70	2.117	0.038
	Control group	2.13 ± 0.74	2.14 ± 0.78	0.052	0.959
	t^*^	0.053	2.072		
	P^*^	0.958	0.043		
Sensitivity	Intervention group	2.06 ± 0.60	1.70 ± 0.59	2.051	0.045
	Control group	2.04 ± 0.60	2.01 ± 0.60	0.197	0.845
	t^*^	0.131	2.051		
	P^*^	0.896	0.045		
Depression	Intervention group	1.93 ± 0.65	1.51 ± 0.61	2.374	0.021
	Control group	1.92 ± 0.63	1.84 ± 0.63	0.625	0.534
	t^*^	0.062	1.51 ± 0.61		
	P^*^	0.951	1.84 ± 0.63		
Anxiety	Intervention group	1.91 ± 0.56	1.52 ± 0.53	2.166	0.034
	Control group	1.85 ± 0.57	1.83 ± 0.56	0.836	0.406
	t^*^	0.418	2.239		
	P^*^	0.677	0.029		
Hostility	Intervention group	1.72 ± 0.57	1.51 ± 0.43	2.162	0.036
	Control group	1.74 ± 0.55	1.73 ± 0.41	0.073	0.942
	t^*^	0.141	2.062		
	P^*^	0.889	0.044		
Phobia	Intervention group	1.65 ± 0.41	1.55 ± 0.38	0.996	0.323
	Control group	1.61 ± 0.41	1.57 ± 0.42	0.379	0.706
	t^*^	0.384	0.197		
	P^*^	0.702	0.845		
Paranoia	Intervention group	1.62 ± 0.55	1.41 ± 0.50	1.573	0.121
	Control group	1.68 ± 0.53	1.65 ± 0.54	0.221	0.826
	t^*^	0.437	1.816		
	P^*^	0.663	0.074		
Psychosis	Intervention group	1.35 ± 0.43	1.29 ± 0.36	0.596	0.554
	Control group	1.34 ± 0.42	1.31 ± 0.41	0.285	0.777
	t^*^	0.093	0.204		
	P^*^	0.927	0.839		

* Represents cross-group comparison,

# represents inter-group comparison

## Discussion

### Analysis of Mental Health Status of Medical Workers

In this study, 801 medical workers were examined using the SCL-90. The results showed that their average SCL-90 score, number of positive items, and scores on the factors of obsessive-compulsive symptoms, sensitivity, depression, anxiety, and phobia, were significantly higher than the national norm (*P* < 0.05), while there was no significant difference from the norm in scores score on somatization, hostility, paranoia, or psychosis (*P* > 0.05). Overall, medical workers’ mental health levels were lower than the national norm. The results are partially consistent with earlier studies (17–19), where medical workers’ overall SCL-90 scores and scores on all dimensions were significantly higher than the national norm. Meanwhile, Gu et al. (20) investigated medical works in general hospitals in Jiangsu Province and found that medical workers did not differ significantly from the national norm on somatization, consistent with the results of this study.

The consistency among the relevant current research on the lower overall mental health levels of medical workers suggests that the mental health of medical workers should cause social attention and concern. The inconsistencies in some specific findings suggest that there are complicated factors influencing the mental health of medical workers. Most investigators have used the SCL-90, widely used to measure individual mental health, to examine mental health of medical workers, ensuring comparability between these findings. However, Wang et al. (17) focused on medical workers in Beijing tertiary general hospitals, Hu et al. (18) surveyed pediatricians in Hainan Province, and Ji et al. (19) investigated medical workers in Qingdao tertiary general hospitals. Beijing, Hainan, and Qingdao are all coastal cities and economically developed areas with large populations; medical workers there are faced with fierce internal competition, large workloads, and more prominent differences between doctors and patients. In contrast, Gu et al. (20) explored the mental health levels of medical workers in Jiangsu Province. Compared to more developed areas, Jiangsu Province and the province of the present study are both characterized by smaller hospital competition pressure and survival pressure, which may explain the inconsistencies in the results. Another possible cause is related to the sample size and the nature of the sample. Wang (17) surveyed 2460 participants, but only 405 participants were involved in the study by Hu et al. (18). Meanwhile, some studies investigated all departments in a hospital, while others only investigated a particular department. Thus, whether the samples of different studies can be compared needs to be further demonstrated.

### The Intervention Effect of the Exercise*–*Psychology Adjustment Mode on the Mental Health of Medical Workers

There was no significant difference between the intervention group and the control group in SCL-90 score, number of positive items, or score on each factor before the intervention (*P* > 0.05). After the intervention, the SCL-90 score, number of positive items, and scores on somatization, obsessive-compulsive symptoms, sensitivity, depression, anxiety, and hostility of the intervention group were significantly improved (*P* < 0.05) and lower than those of the control group (*P* < 0.05). The scores of the intervention group on phobia, paranoia, and psychosis were also lower than those of the control group, but the differences were not significant (*P* > 0.05).

Overall, the exercise–psychology adjustment mode is an effective method for intervening in the mental health of medical workers. Research on physical exercise and mental health has shown that individuals who regularly participate in physical exercise have significantly higher mental health levels and longer lifespans than those who do not often partake in physical exercise (21). This provides support for the empirical study of the application of the exercise–psychology adjustment mode. Zhang (12) first used the exercise–psychology adjustment mode to conduct mental a health intervention on 20 medical students within 12 weeks, under an intervention procedure similar to that of this study, and found that the intervention had a significantly positive and lasting effect on medical students’ self-promotion and self-adjustment of mental health. Zhang (22) used a nonequivalent intervention and control group experimental design to study the effect of mental intervention on the mood and well-being of psychiatrists, with 30 psychiatric workers and 34 ordinary people in each group. After the mental intervention, those psychiatric workers had significantly higher levels of mental health, lower levels of depression and anxiety, and increased psychological well-being. Nonetheless, compared with ordinary people, their mental health level was still at a low level. This study showed the necessity of carrying out mental interventions in medical workers. To improve the mental health levels of medical workers, it is critical to analyze the factors affecting their mental health from multiple angles and aspects, and to develop targeted intervention measures.

Mental intervention improved the mental health of medical workers, but the intervention effect neither lasted long nor made the mental health level of medical workers equal to that of ordinary people (22). This to some extent indicates the limited effect of mental intervention. In comparison, the exercise–psychology adjustment mode could achieve a relatively ideal intervention effect. This is due to the essential characteristics of the two intervention approaches: Mental intervention emphasizes the psychological problems of the subjects and employs relevant theories and techniques of psychotherapy to treat systematically the subjects (23). This intervention is often dominated not by the subjects but by psychotherapists with rich clinical experience. Consequently, in this intervention, the subjects are in a passive state. Additionally, conventional mental intervention is usually performed in a narrow context. Once the environment is changed, the effect of intervention is greatly reduced. In contrast, the exercise intervention mode has unique advantages. First, physical exercise can vary from person to person; each person can choose the appropriate form of physical exercise, exercise load, and exercise time according to their specific psychological problems and physical conditions (24). Second, physical exercise can give full play to the initiative of the individuals, leading them to enhance gradually self-confidence in the exercise and to experience subtle changes in their attitudes (25). Third, due to the cross-context applicability of physical exercise, medical workers facing heavy work pressure and long work hours can choose different forms of exercise in different environments such as the office and home, likely resulting in a better effect of exercise intervention.

Nonetheless, our study had some limitations, and further research on the subject is still necessary. Firstly, this study used SCL-90, other mental health tests with higher reliability and higher recognition could be used to investigate and enrich relevant data in the future. Secondly, if conditions permit, the scope and subjects of the survey should be expanded as much as possible, perhaps to a nationwide or a large-scale regional census. Future research could also compare the mental health status of medical workers in the same department or the same job position in different areas so as to increase the comparability between different studies and refine relevant influencing factors such as working environment, workload, and so on. Finally, this study only distinguished between the medical workers participating in the investigation according to their SCL-90 score and related scores, making no detailed distinction between the different subjects in terms of main psychological problems, work department, workload, and so on. Although we did choose and plan the exercise mode and exercise load in accordance with the characteristics of each subject before the intervention, future studies could take into account the various background information of the subjects, such as work department, to improve the relevance of the intervention and thereby enhance the intervention effect.

## Conclusion

After the exercise–psychology adjustment mode was used to intervene in the mental health of medical workers with positive SCL-90 results, their mental health was significantly improved, as reflected by the significant improvement in SCL-90 score, number of positive items, and scores on somatization, obsessive-compulsive symptoms, sensitivity, depression, anxiety, and hostility. Meanwhile, scores on phobia, paranoia, and psychosis did not differ significantly from those before the intervention, but improved to some extent. Thus, exercise–psychology adjustment is effective at improving medical workers’ mental health level.

In conclusion, the mental health of medical workers is an important problem facing the society at present. The exercise–psychology adjustment mode has the advantage of being economical and easy to popularize, and can therefore be used as an effective means of intervention in the mental health of medical workers.

## Ethical considerations

Ethical issues (Including plagiarism, informed consent, misconduct, data fabrication and/or falsification, double publication and/or submission, redundancy, etc.) have been completely observed by the authors.
